# Measurement of CeO_2_ Nanoparticles in Natural Waters Using a High Sensitivity, Single Particle ICP-MS

**DOI:** 10.3390/molecules25235516

**Published:** 2020-11-25

**Authors:** Ibrahim Jreije, Agil Azimzada, Madjid Hadioui, Kevin J. Wilkinson

**Affiliations:** 1 Biophysical Environmental Chemistry Group, University of Montreal, P.O. Box 6128, Succ. Centre-Ville, Montreal, QC H3C 3J7, Canada; ibrahim.jreije@umontreal.ca (I.J.); agil.azimzada@mail.mcgill.ca (A.A.); Madjid.hadioui@umontreal.ca (M.H.); 2 Department of Chemical Engineering, McGill University, Montreal, QC H3C 3J7, Canada

**Keywords:** nanoparticles, cerium oxide, single particle ICP-MS, sector field, time of flight, natural waters

## Abstract

As the production and use of cerium oxide nanoparticles (CeO_2_ NPs) increases, so does the concern of the scientific community over their release into the environment. Single particle inductively coupled plasma mass spectrometry is emerging as one of the best techniques for NP detection and quantification; however, it is often limited by high size detection limits (SDL). To that end, a high sensitivity sector field ICP-MS (SF-ICP-MS) with microsecond dwell times (50 µs) was used to lower the SDL of CeO_2_ NPs to below 4.0 nm. Ag and Au NPs were also analyzed for reference. SF-ICP-MS was then used to detect CeO_2_ NPs in a Montreal rainwater at a concentration of (2.2 ± 0.1) × 10^8^ L^−1^ with a mean diameter of 10.8 ± 0.2 nm; and in a St. Lawrence River water at a concentration of ((1.6 ± 0.3) × 10^9^ L^−1^) with a higher mean diameter (21.9 ± 0.8 nm). SF-ICP-MS and single particle time of flight ICP-MS on Ce and La indicated that 36% of the Ce-containing NPs detected in Montreal rainwater were engineered Ce NPs.

## 1. Introduction

Due to their unique properties, engineered nanomaterials are now widely used in numerous commercial products. Cerium oxide (CeO_2_) nanoparticles (NPs) are among the most commonly used engineered NPs, with applications in catalysis [1,2,3], the manufacturing of semiconductors [2], biomedicine [4] and agriculture [5], among other fields. For example, they are commonly found as UV filters in sunscreens, additives in diesel fuels and as a component of paints and stains [6,7]. With the significant increase in the production and use of CeO_2_ NPs, concern is increasing over their release into the environment and their subsequent fate and toxicity. Among their important environmental pathways, CeO_2_ NPs can be released into the air by diesel emissions; into soils from solid waste and recycling and into aquatic systems from the effluents of wastewater treatment plants [1]. Furthermore, a number of recent papers [8,9,10] have measured the release of CeO_2_ NP from surface coatings such as paints and stains. Nonetheless, the vast majority of data on the concentrations, fate and transformations of CeO_2_ NP in the environment have been generated by modelling [11,12], or extrapolated from release studies performed under controlled laboratory conditions. This is mainly because the direct analysis of CeO_2_ NPs in the environment is extremely challenging due to their small sizes (often below 20 nm), their low concentrations (on the order of ng L^−1^) and the complexity of environmental matrices, which also contain natural colloids.

Techniques based upon inductively coupled plasma mass spectrometry (ICP-MS) are likely to be the most promising for detecting and quantifying inorganic NP in biological and environmental samples. Particle size distributions can be obtained either by coupling a separation technique such as field flow fractionation (FFF) [13] or hydrodynamic chromatography (HDC) [14] upstream of the ICP-MS or by performing ultrafast measurements on single particles (single particle (SP) ICP-MS). SP-ICP-MS combines the specificity and sensitivity of mass spectrometry with a particle-by-particle analysis, enabling quantification of very low NP concentrations (ng L^−1^) and information on their number concentrations, sizes, and size distributions. Transient ion clouds that are created from the NP in the plasma (typically 300–500 µs) are directly related to the particle number concentrations, while the signal intensity is related to particle (elemental) mass [15]. For a given NP composition, density and geometry, particle size can be calculated.

The very small NPs are expected to have a greater environmental risk, due to their increased propensity to cross biological membranes [16,17]. Therefore, it is especially important to obtain rigorous size, concentration, and fate data for the smallest NP. The minimal particle size (size detection limit = SDL) that can be measured by SP-ICP-MS depends largely upon the signal to noise ratio. A number of approaches have been employed to decrease the SDL [18] including: sample dilution, judicious choice of isotopes, interference removal strategies [19,20], shorter dwell times [21], etc.

The first objective of this study was to decrease the SDL for CeO_2_ NPs using a high sensitivity sector field ICP-MS (SF-ICP-MS), very short dwell times (<100 µs) and the introduction of a dry aerosol. The optimized method was then used to detect, quantify, and characterize CeO_2_ NPs in several natural water samples.

## 2. Results and Discussion

### 2.1. Optimization of SP-ICP-MS for CeO_2_ NPs

**Figure 1:**
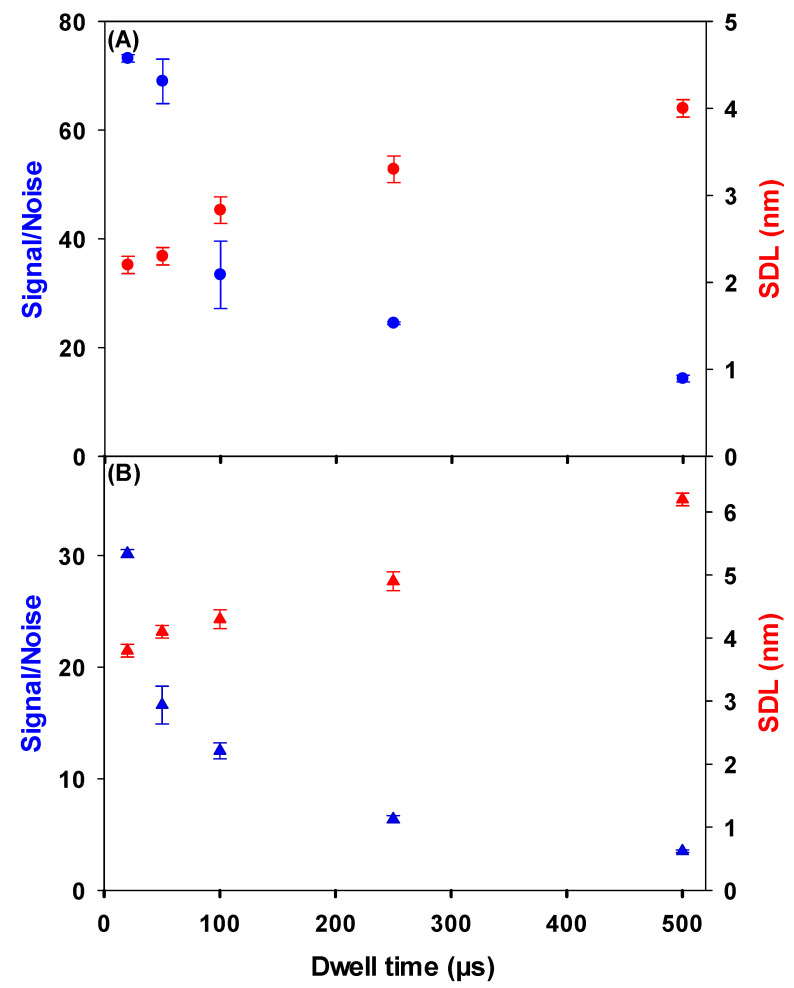
Signal: Noise (blue) and size detection limits (red) as a function of dwell time for: (**A**) a 10 ng L^−1^ suspension of CeO_2_ NPs (1–10 nm) in Milli-Q water; (**B**) a 10 ng L^−1^ suspension of CeO_2_ NPs (1–10 nm) in a Milli-Q water containing 10 ng L^−1^ of ionic Ce. Signal/Noise was determined by dividing the average intensity of a well resolved peak of a 9 nm CeO_2_ NP by the average intensity of the continuous background. All measurements were obtained using SF-ICP-MS and sample introduction via a desolvator.

In single particle ICP-MS, size detection limits (SDL) are minimized for Ce NPs by optimizing the signal/noise (S/N) for ^140^Ce^+^. In this case, noise includes both electronic noise and background concentrations, comprised of isobaric and polyatomic interferences, dissolved Ce and Ce NPs that are smaller than the SDL. The impact of dwell times on the SDL [21] was first tested on a suspension of CeO_2_ NPs in Milli-Q water, with or without dissolved Ce. While both spike intensity and background signal decreased as dwell time was decreased from 500 to 20 µs (Figure S1), S/N was clearly improved at the shorter dwell times (Figure 1). For example, for a decrease in dwell time from 500 µs to 20 µs, the SDL decreased from 4 nm to 2.2 nm in Milli-Q water (Figure 1A) and from 6.2 nm to 3.8 nm when the data were acquired in the presence of 10 ng L^−1^ of dissolved Ce (Figure 1B). Although the lowest SDL was measured with a 20 µs dwell time, it was not used further here since analysis was limited to one million datapoints per run (and thus an overall analysis time of 20 s). For environmental samples, longer acquisition times better ensure representativity by better taking into account sample polydispersity and low particle numbers. Given the slight difference in SDL between dwell times of 20 and 50 µs (Figure 1), a dwell time of 50 µs and a corresponding 50 s acquisition time were used in a majority of subsequent experiments.

Sensitivity and transport efficiency were determined for gold, silver and CeO_2_ NPs using a quadrupole based ICP-MS (Q-ICP-MS) and a SF-ICP-MS, using wet or dry (i.e., desolvator) sample introduction. Sensitivity increased about 100× for Ag and 200× for Au and Ce when using the SF-ICP-MS as compared to the Q-ICP-MS (Table 1). This increase of sensitivity led to a lowering of the SDL from 16.3 to 4.7 nm for Ag; from 19.0 to 4.4 nm for Au and from 16.8 to 4.0 nm for CeO_2_. For the Q-ICP-MS, similar SDL have previously been reported for CeO_2_ in the literature [7,22]. When the sample was introduced as a dry aerosol, sensitivity increased a further 5× for Ag and Au and 3× for Ce, resulting in a lowering of the SDLs to 3.1 nm (Ag), 2.8 nm (Au) and 2.3 nm (CeO_2_) (Table 1).

**Figure 2:**
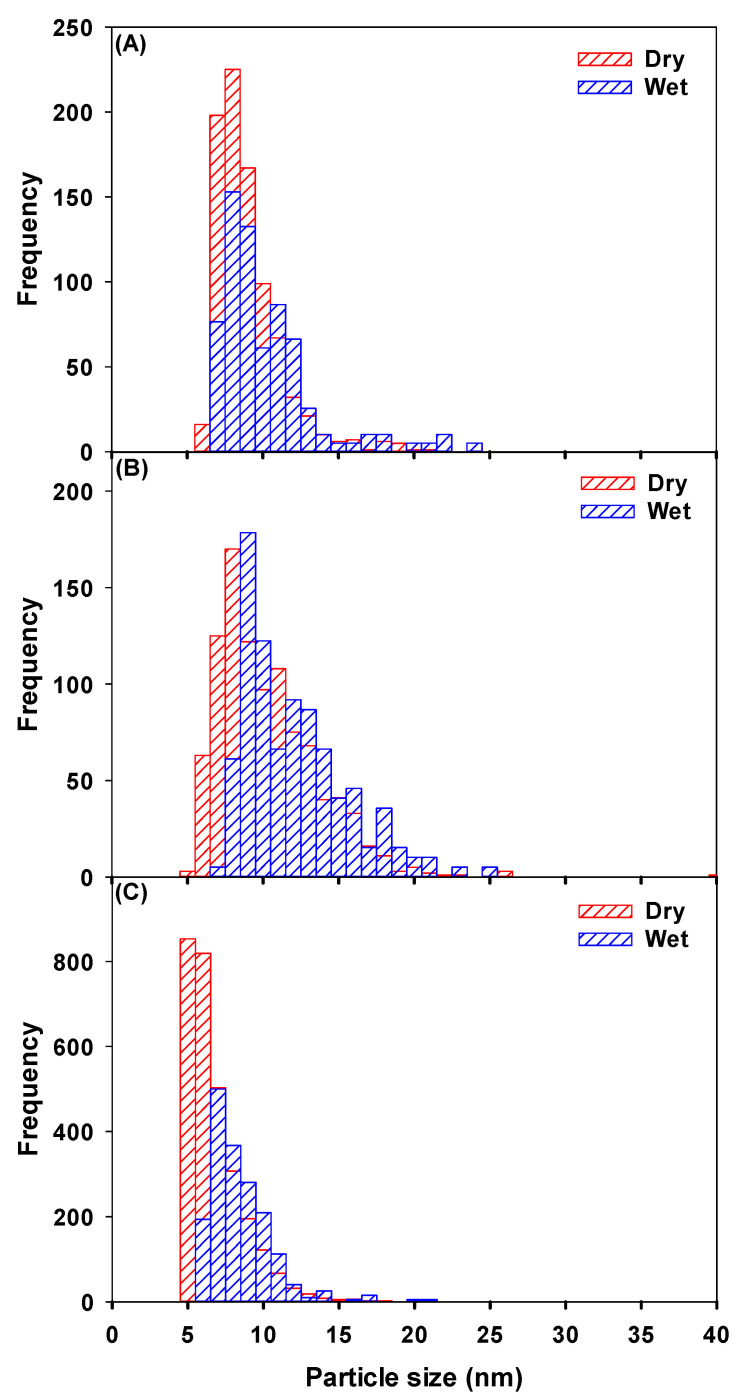
Particle size distributions as measured by wet-SF-ICP-MS (blue) and dry-SF-ICP-MS (red) for 3 suspensions of a small NP: (**A**) Ag: the mean particle size ± polydispersity of the suspension was 8.5 ± 2.2 with dry introduction and 9.8 ± 3.3 with wet introduction; (**B**) Au: the mean particle size ± polydispersity of the suspension was 9.8 ± 3.4 with dry introduction and 11.8 ± 3.6 with wet introduction; (**C**) CeO_2_: the mean particle size ± polydispersity of the distribution in the suspension was 6.3 ± 2.0 with dry introduction and 8.0 ± 2.1 with wet introduction.

The role of membrane desolvation was further evaluated by comparing size distributions and particle number concentrations for small Ag, Au and CeO_2_ NPs (nominally 10 nm Ag, 10 nm Au and 1–10 nm CeO_2_), using both dry and wet aerosol introduction (Table 2). Some smaller NPs were detected using the dry-SP-ICP-MS due to the lower SDL, consequently leading to higher particle number concentrations (approximately 1.5× more NPs when compared to wet aerosol injection) and a lower average particle size (Figure 2).

The difference between the two setups became more significant for an increasing proportion of dissolved metal in the suspensions, which reversed the impact of the desolvator. For example, when a 10 ng L^−1^ suspension of CeO_2_ NPs was spiked with 10 ng L^−1^ of dissolved Ce, particle size distributions were similar, with similar average sizes (8.1 ± 0.1 nm for dry-SF-ICP-MS and 8.3 ± 0.1 nm for wet-SF-ICP-MS) (Figure S2A), however, nearly 1.6× more CeO_2_ NPs were detected using wet-SF-ICP-MS as compared to dry-SF-ICP-MS. Moreover, when a 10 ng L^−1^ suspension of CeO_2_ NPs was spiked with 50 ng L^−1^ of dissolved Ce, significantly different particle number concentrations were measured with the two introduction systems with nearly 5× more CeO_2_ NPs detected by wet-SF-ICP-MS (Figure S2B). It would appear that when using the desolvator, both the background signal and the NP signal increased, however, background increased to a greater proportion. Therefore, in the presence of significant dissolved metal, the S/N decreased when using dry-SF-ICP-MS as compared to the wet-SF-ICP-MS (Figure S3). For example, for a 9.0 nm CeO_2_ NP combined with 50 ng L^−1^ of dissolved Ce, the S/N was 2.8 ± 0.3 when using dry-SF-ICP-MS and 6.8 ± 0.1 when using wet-SF-ICP-MS. When NP suspensions containing increasing dissolved Ce were analyzed in single particle SF-ICP-MS, results clearly showed that the desolvator was more sensitive to the presence of the dissolved fraction with higher SDLs as compared to the wet-SF-ICP-MS (Figure S4). Therefore, although smaller SDLs could be determined by dry-SF-ICP-MS for very low concentrations of dissolved Ce; due to an unknown and variable proportion of dissolved Ce, the introduction of a wet aerosol was prioritized for natural samples.

### 2.2. Ce-Containing NPs in Natural Waters: Rainwater

**Figure 3:**
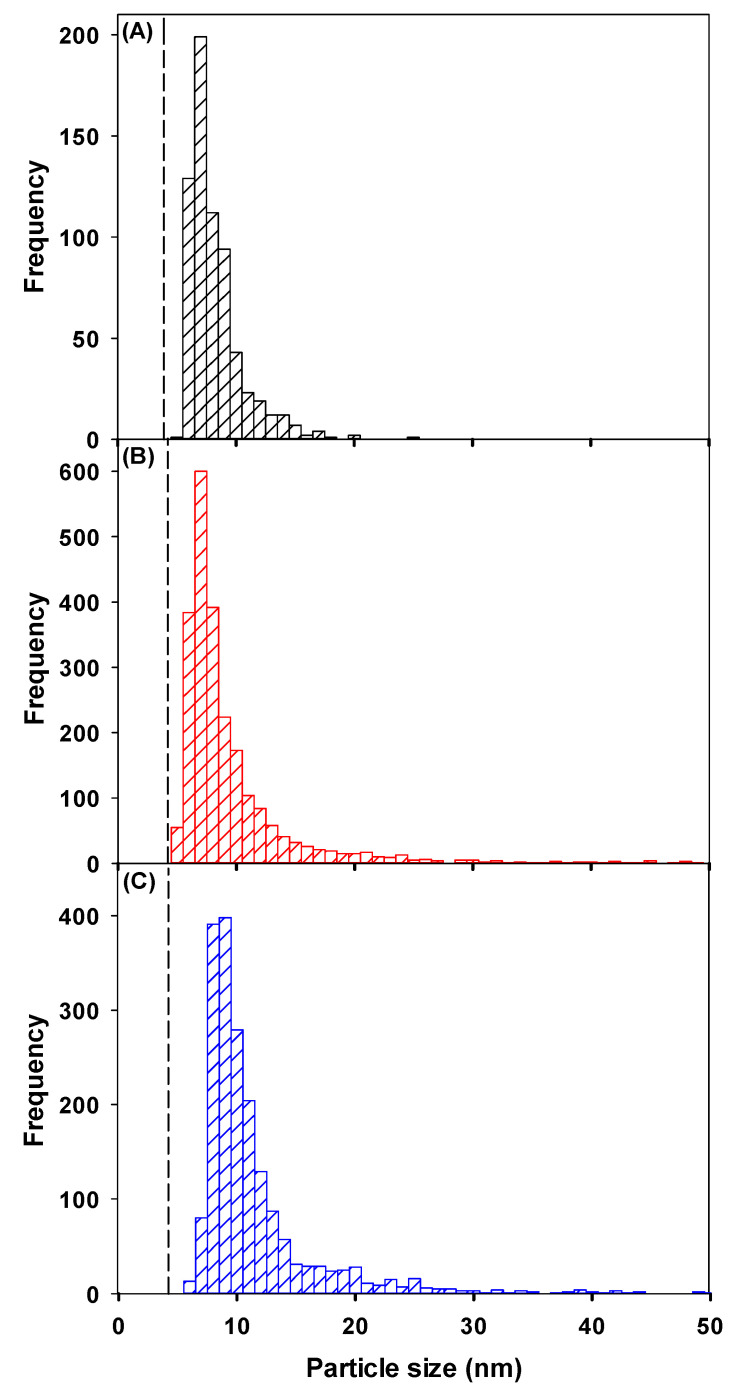
Particle size distribution of a suspension of CeO_2_ NPs with a nominal size of 1–10 nm. NP were spiked into (**A**) Milli-Q water or (**B**) rainwater. (**C**) Particle size distribution for Ce-containing NPs in rainwater, determined under the assumption that they were spherical CeO_2_ NPs with a density of 7.13 kg dm^−3^ [23]. The dashed line corresponds to the SDL. The mean particle size ± polydispersity of the CeO_2_ NPs was (**A**) 11.0 ± 5.8 nm; (**B**) 7.7 ± 2.4 nm and (**C**) 9.2 ± 6.1 nm. Measurements were obtained using the wet-SF-ICP-MS with a 50 µs dwell time.

The wet-SF-ICP-MS was first used to determine whether Ce NP could be detected in rain. In the rainwater matrix alone, significant numbers of Ce-containing NPs were found, with a mean particle diameter of 10.8 ± 0.2 nm (Figure 3C, calculated under the assumption that the particles were spherical CeO_2_ NPs). Subsequently, 10 ng L^−1^ of 1–10 nm CeO_2_ NPs were spiked into the rainwater and compared with observations performed in Milli-Q water. An average particle diameter of 7.9 ± 0.2 nm was determined in Milli-Q water (Figure 3A), which was smaller than the size obtained for the NP spiked into rainwater (9.5 ± 0.4 nm, Figure 3B). About three times more CeO_2_ NPs were detected in the spiked rainwater (5.1 × 10^8^ NPs L^−1^) as compared to the spiked Milli-Q water (1.6 × 10^8^ NPs L^−1^), which was reasonable given the initial NP numbers found in the unspiked rainwater (3.8 × 10^8^ NPs L^−1^). These values corresponded to a recovery for the spike of 84 ± 16%. The slightly smaller than expected recovery could be attributed to an increased aggregation of CeO_2_ NPs in the natural precipitation (increased ionic strength), as compared to the Milli-Q water. Note that recovery of total Ce in the rainwater, determined on acidified samples, was 78 ± 24%.

### 2.3. Ce-Containing NPs in Natural Waters: Riverwater

**Figure 4:**
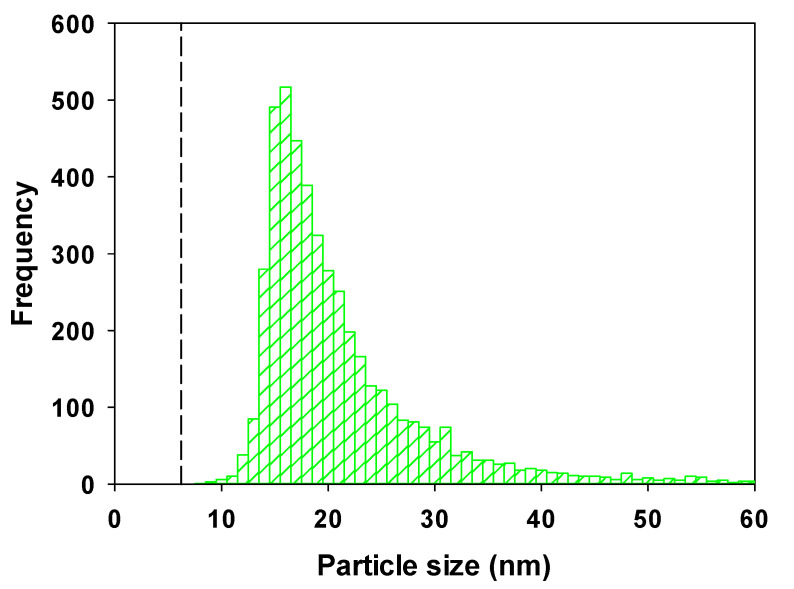
Particle size distributions of Ce containing NPs in a sample taken from the St. Lawrence River. The mean particle size ± polydispersity corresponded to 21.0 ± 9.2 nm. The dashed line corresponds to the SDL. Measurements were obtained using the wet-SF-ICP-MS with a 50 µs dwell time. NP sizes were calculated by assuming that the particles were spherical CeO_2_ particles with a density of 7.13 kg dm^−3^ [23].

When unspiked, unfiltered and undiluted St. Lawrence River water samples were analyzed by SP-ICP-MS for ^140^Ce, the raw data clearly showed the presence of spikes, strongly indicating the presence of Ce containing NPs or, alternatively, larger particles with a minor Ce component (Figure S5). Under the assumption that the detected particles correspond to spherical CeO_2_ NPs, particle size distributions could be estimated (Figure 4). An average particle size of 21.9 ± 0.8 nm was determined for particles sampled from the Saint Lawrence River (means and errors correspond to triplicate samples) (Table 3). The larger particle size, as compared to the rainwater sample could be attributed to both analytical and geochemical differences, i.e., (i) the hardness ions in the St. Lawrence River (~130 mg L^−1^) [24] are expected to induce some agglomeration of the NP; (ii) the lower pH of the rainwater (pH = 5.4), with respect to St. Lawrence River (pH = 6.8), could facilitate greater particle dissolution and (iii) the higher dissolved (background) Ce in the river water sample resulted in a higher SDL (6.2 nm, Table 3), which could slightly increase the overall average particle size. Note that ~7× more NPs and ~60× more particle mass was determined in the river water with respect to the rainwater. Ce NP concentrations in the river water were 107 ± 26 ng L^−1^ (Table 3), in line with concentrations predicted by modelling (24 ng L^−1^, [25]; 0.6–100 ng L^−1^, [26]) and the limited available experimental data (0.4–5.2 ng L^−1^, [27]). While it might be tempting to attribute the slightly higher concentrations of Ce NP to greater emissions in the St. Lawrence, it is more likely that additional NP were detected due to the lower detection limits in this study. Nonetheless, for the majority of detected NP, similar particle size distributions were determined in the St. Lawrence River (mean diameter ~19 nm; Figure 4), as were observed in the Meuse and IJssel Rivers in the Netherlands (14–21 nm, mean 19 nm) [27]). Such a narrow distribution of NP in such geographically different samples suggests either a similar source for the NP or similar removal processes such as agglomeration leading to removal of the larger particles [28,29].

### 2.4. Effect of Sample Filtration

Although the natural samples can be analyzed unfiltered, some of the larger particles can block the nebulizer. Given that we were interested in quantifying the smallest (nano) particles, the effect of 0.45 µm membrane filtration was examined for a natural sample. For a St. Lawrence River water sample examined before or after filtration, filtration decreased background Ce concentrations by 30%, which was attributed to an adsorption of dissolved Ce on the filters. The decrease of dissolved Ce led to a decrease in the SDL, which may explain the small shift of the particle size distribution to lower sizes (Table S1 and Figure S6). Nonetheless, in spite of having a lower SDL, NP numbers decreased almost by 30% after filtration, which was attributed mainly to the removal of large particles (i.e., >0.45 µm) with some minor losses of Ce and/or small Ce-containing NPs to the filters via adsorption.

### 2.5. Ce-Containing NPs in Natural Waters: Temporal Variations

**Figure 5:**
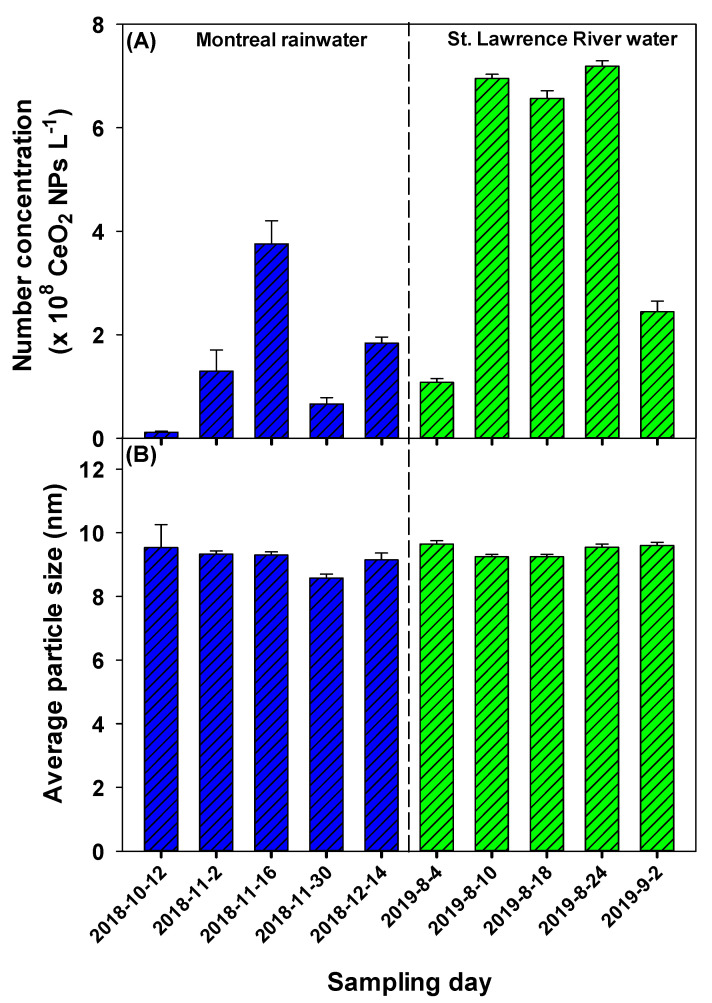
(**A**) Number concentrations and (**B**) mean particle sizes for CeO_2_ NPs measured on different dates in rainwater (blue) and St. Lawrence River water (green). Error bars correspond to standard deviations obtained from triplicate analysis. Measurements were performed using wet-SF-ICP-MS and a dwell time of 50 µs.

Temporal variations of the Ce NPs were examined by collecting rainwater samples between October 2018 and December 2018 and river water samples between August 2019 and September 2019. In the rainwater, the concentration of CeO_2_ NPs varied from (0.1–3.8) × 10^8^ L^−1^, while they varied from (1.1–7.2) × 10^8^ L^−1^ in the river water (Figure 5A). Although particle number variations were more important in the rainwater (38×), as compared to the river water (7×), particle sizes were fairly similar in all samples (Figure 5B). In contrast, NP sizes in the river water were smaller than those detected previously in the same water catchment (St. Lawrence), but much further downstream stream (Figure 4). This result can be explained by the difference in sampling locations, which would indicate that the Ce-containing NPs are both time and geographically dependent (Figure S7).

### 2.6. Engineered or Natural CeO_2_ NPs?

It is very difficult to non-ambiguously distinguish between engineered and natural NPs in the environment. Most commonly, it is assumed that engineered NPs (including metal oxides) contain a single metal, while natural NPs are often multi-element [30]. For Ce NP, the Ce/La ratio has been used to differentiate the two types of NPs [31,32] since the rare earth elements are naturally nearly always found as mixtures. Natural NPs can be formed by mechanical erosion, weathering or precipitation [33] in which case, they are likely to have a similar composition as the minerals in sediments and soils. For example, Ce is found with La in minerals such as bastnasite with a Ce/La ratio of 1.5 [1,34] and in monazite with a Ce/La ratio of 1.8 [1,34]. In the earth’s crust, a Ce/La ratio of 2.1 [35] has been documented. In that light, Montaño et al. [30] compared Ce/La ratios of nearly 100 surface water samples collected from three European countries to the Ce/La ratios of 807 water samples collected across a wide geographical range in Europe. In those samples, a fairly stable Ce/La ratio of 1.7 was measured.

In order to determine whether the NP contained only Ce or several metals, SF-ICP-MS was first used to measure individual particles for their Ce and La contents, albeit in different particles. Results were later validated with TOF-ICP-MS, run in single particle mode, which measures multiple elements in a single nanoparticle. The TOF-ICP-MS that was employed had a sensitivity for Ce (597 counts fg^−1^) that was about 8× higher than the Q-ICP-MS (72 counts fg^−1^) but lower than the SF-ICP-MS (14,800 counts fg^−1^) and was necessarily limited to larger nanoparticles (SDL~11 nm).

**Figure 6:**
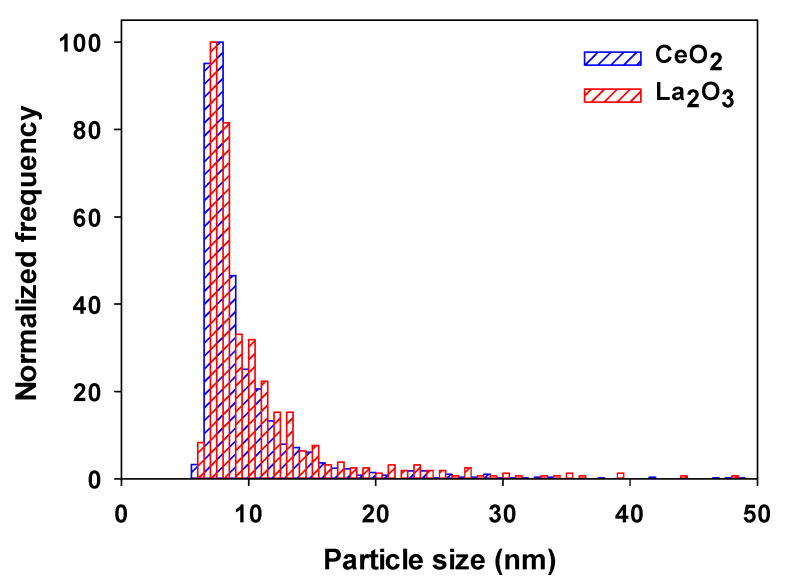
Particle size distributions of Ce- (blue) and La-(red) containing NPs found in a Montreal rainwater. Particle size distributions were calculated by assuming spherical particles of pure CeO_2_ (density = 7.13 kg dm^−3^) [23] and La_2_O_3_ (density = 6.51 kg dm^−3^) [36]. Measurements were obtained using the wet-SF-ICP-MS with a 50 µs dwell time.

The rainwater was analyzed for its Ce and La contents by SF-ICP-MS. Similar sensitivities were measured for La (14,600 ± 1700 counts fg^−1^) and Ce (13,600 ± 500 counts fg^−1^), which led to similar SDLs in the rainwater of 4.4 ± 0.1 nm for La_2_O_3_ and 4.6 ± 0.1 nm for CeO_2_. The raw data clearly indicated the presence of both Ce and La NPs or indeed composite NPs (Figure S8). As a control, no La (dissolved or NP) was detected in the suspension of 1–10 nm CeO_2_ NPs. Detected Ce and La NPs showed similar mean sizes and size distributions (Table S2, Figure 6). Concentrations of both the dissolved and particulate forms of Ce were almost 2× those of La with a Ce/La NP ratio of 2.2 ± 0.6 and a total Ce/La ratio of 2.4 ± 2.2 (Table 4). The slightly higher values, when compared to the expected global natural ratio of 1.7, was suggestive of the presence of engineered (single element) CeO_2_ NPs in the rainwater.

**Figure 7:**
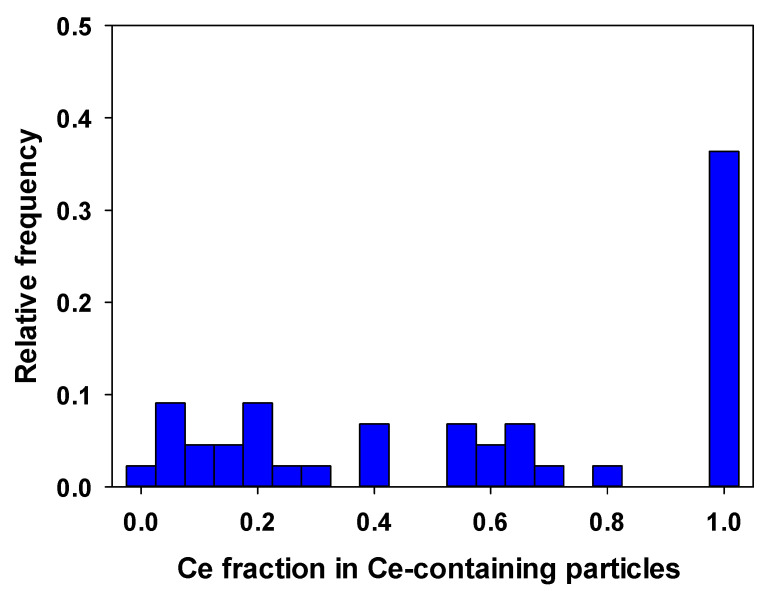
Detected proportion of Ce in Ce-containing particles measured in a Montreal rainwater. A Ce fraction of 1 indicates a pure Ce NP, while a fraction < 1 indicates the presence of other elements in the particles. Measurements were obtained using the TOF-ICP-MS with a 76.5 µs dwell time; SDL (Ce) = 11 nm and SQL (Ce) = 16 nm.

The rainwater sample was re-analyzed by TOF-ICP-MS in single particle mode. Given the size quantification limits (SQLs of ~16 nm for Ce and for La), only few Ce-containing particles were detected in the rainwater (1.5 × 10^6^ L^−1^). Nonetheless, for 36% of the Ce-containing nanoparticles in the rainwater, no second element was detected (Figure 7 and Figure S9).

## 3. Materials and Methods

### 3.1. Engineered NPs

The cerium oxide NPs used in this study were purchased as an aqueous dispersion of citrate stabilized CeO_2_ NPs with a nominal size range of 1–10 nm (Nanobyk^®^-3810, Byk, Weiser, Germany). Silver and gold NPs were used to determine transport efficiency and validate instrumental sensitivity and precision. Ag NPs were purchased from NanoComposix (San Diego, CA, USA) as a citrate stabilized suspension with nominal sizes of either 10 nm (NanoXact, AGCN10) or 20 nm (NanoXact, AGCN20). Three Au NPs were used. One was purchased as citrate stabilized suspension from NanoComposix (10 nm, NanoXact, AUCN10), a second was a monodisperse poly(ethylene glycol) carboxylated Au NP (30 nm, UltraUniform, AUXU30, NanoComposix) and a third was acquired from the National Institute of Standards and Technology (60 nm, NIST, SRM 8013, Gaithersburg, MD, USA).

NP stock suspensions were stored in the dark at 4 °C until use. Prior to analysis, the stock suspensions were vortexed for one minute, sonicated for 10 min (Branson Ultrasonic Cleaner, 5510R-DTH Model, 135 W), diluted to adequate concentrations in Milli-Q water (R > 18.2 MΩ cm; total organic carbon < 1 μg L^−1^), and then re-vortexed for one minute. Final suspensions had a mass concentration between 10–50 ng L^−1^, depending on the NP size. Concentrations were optimized in order to ensure a statistically significant number of events, while reducing the probability for the concurrent atomization and ionization of more than one NP (which would lead to an overestimation of particle size and underestimation of particle number). To evaluate the impact of background noise on NP size and concentration determinations, a 10 ng L^−1^ suspension of the Nanobyk^®^ CeO_2_ NPs was spiked with 5–100 ng L^−1^ of ionic Ce purchased from Inorganic Ventures (CGCE1, Christianburg, VA, USA).

The total Ce concentration was determined by adding 400 µL of HNO_3_ (67–70%) and 300 µL of H_2_O_2_ (30%) to 1 mL of the sample, prior to heating the mixture at 80 °C for 5 h (DigiPREP, SCP science, Montreal, Canada). Samples were then diluted to 2% *v*/*v* HNO_3_ prior to their analysis by ICP-MS, using the ionic Ce (CGCE1) for calibration and indium (CGIN1) as an internal standard. Ce and In standards were purchased from Inorganic Ventures.

### 3.2. Sampling and Sample Preparation

Rainwater samples were collected, between October and December 2018, using wide-mouth polypropylene containers (500 mL, Fisher Scientific), which were placed on the 7th floor roof of the Roger-Gaudry Pavillon of the University of Montreal (Montreal, QC, Canada). St. Lawrence River water samples were collected from two different locations (Figure S7), between August and September 2019, using polypropylene tubes (50 mL, Fisher Scientific), at a depth of 20–30 cm, 1 m from the shore. All samples were stored at 4 °C in the dark. Prior to their analysis, water samples were first shaken manually, sonicated for 10 min, vortexed for one minute and then filtered through a 0.45 µm, 33 mm diameter PVDF syringe filter. Filters were pre-rinsed with Milli-Q water and 5 mL of sample.

### 3.3. Instrumentation

Single particle ICP-MS data was acquired in fast scan mode using a quadrupole ICP-MS (Q-ICP-MS; Perkin Elmer NexION 300×, Woodbridge, ON, Canada) or a double focusing magnetic sector field ICP-MS (SF-ICP-MS; Nu AttoM ES, Nu Instruments, Wrexham, UK). The introduction system for the Q-ICP-MS consisted of a quartz cyclonic spray chamber, a type C0.5 concentric glass nebulizer (0.5 mL min^−1^) and a quartz 2 mm bore injector. In the case of the SF-ICP-MS, two introduction systems were used: (i) A micro-flow concentric glass nebulizer (self-aspiration rate of 200 µL/min for 1 L min^−1^ argon gas) with a quartz cyclonic spray chamber cooled to 4 °C (wet-SF-ICP-MS); or (ii) an Apex Omega desolvator (Elemental Scientific, Omaha, NE, USA). When using the desolvator (dry-SF-ICP-MS), the sample was nebulized, with a PFA (perfluoroalkoxy) concentric micro-flow nebulizer (self-aspiration rate of 200 µL min^−1^) in a quartz cyclonic spray chamber that was heated to 140 °C before condensation at 3 °C and passage through a porous PFA membrane heated at 160 °C. Argon was used as the membrane sweep gas (6–8 L min^−1^) and as the nebulizer gas (0.7–1.0 L min^−1^). In both introduction systems, the aerosol was injected into the plasma through a 1.5 mm internal diameter quartz injector.

Time of flight ICP-MS (TOF-ICP-MS) measurements were performed on a time-of-flight ICP-MS (Vitesse, Nu Instruments, Wrexham, UK) that allowed for multi-element characterization of individual nanoparticles. The instrument used a segmented reaction cell in which 4–6 cm^3^ min^−1^ of He and ca. Four cm^3^ min^−1^ of H_2_ gas was introduced in order to eliminate argon and nitrogen-based interferences for elements such as Si, K, Ca, Cr and Fe [37]. Time of flight mass spectra (23–238 amu) were acquired with a dwell time of 76.5 µs [37]. Total sample acquisition time was 32 s. In some experiments, the instrument was coupled to an Aridus II desolvator (Teledyne Cetac Technologies, Omaha, NE, USA) (Argon was used as the membrane sweep gas (5–6 L min^−1^) and as the nebulizer gas (0.9–1.0 L min^−1^)). Calibrations were performed using different custom-prepared standards (CLMS-1, CLMS-2, CLMS-3 and CLMS-4, SPEX CertiPrep, Metuchen, NJ, USA) that included all of the metals and metalloids.

### 3.4. SP-ICP-MS Data Acquisition

Triplicate samples were each analyzed three times- means and standard deviations were determined from the triplicate samples and triplicate measurements. In addition, in some cases, the breadth of the particle size distributions was indicated by calculating polydispersities. The isotopes ^107^Ag, ^197^Au, ^139^La and ^140^Ce were measured using a resolution of 300 and a dwell time of 50 µs. Data were acquired for 50 s at a sample flow rate of 100–200 µL min^−1^. External calibrations (0.05 to 1.0 µg L^−1^) were performed using ionic standards (Ag, La and Ce: Inorganic Ventures; IV-ICP-MS-71A; Au: Inorganic Ventures; MSAU-100PPM, Christianburg, VA, USA). Sensitivities were validated with an ionic quality control standard provided by High-Purity Standards (QCS-27). Transport efficiency (TE) was determined [38] by measuring the instrument sensitivity for ionic Au standards and the particle number concentration of a standard suspension of Au NPs. For analysis with the Q-ICP-MS, a 50 ng L^−1^ suspension of 60 nm Au NPs (NIST) was used for TE determinations, while for SF- and TOF-ICP-MS, a suspension of ultra-uniform 30 nm Au NPs (NanoComposix) was prepared daily at 20 ng L^−1^. Furthermore, TE was validated by verifying the sizes of Ag NPs (NanoComposix) with a nominal size of 20 nm. TE values for the Q-ICP-MS were between 3–5%. With SF-ICP-MS, they were between 4–7%, except when using the desolvator (15–20%). For the TOF-ICP-MS, TE values ranged between 10–15%.

### 3.5. SP-ICP-MS Data Processing

Q-ICP-MS data were processed using the Syngistix Nano Module (Perkin Elmer, Woodbridge, ON, Canada). Peaks were considered if their intensity was greater than the average background + three times the standard deviation of the background. SF-ICP-MS data were processed using Nu Quant software (version 2.2, Nu Instruments, Wrexham, UK [18,39]). A built-in algorithm searches in a fixed window (3–15 ms) for a peak maximum that is greater than the signal of the smoothed background. When a maximum is found, the algorithm searches for the pre- and post-inflection points, integrates the data between these points and subtracts local peak background. Local peak background is determined from smoothed data prior to the pre-inflection point of the peak. The script also calculates the full width half maximum (FWHM) for each peak, which was used on several occasions to identify artifacts and coincidence (overly large FWHM). A major difference between the programs is that the Syngistix module identifies peaks with respect to the average signal background, whereas Nu Quant employs the local background. In both cases, the concentration of dissolved metal is determined from the average background signal, obtained from the entire data set.

Size detection limits (nm) are determined from the threshold intensity (*I*_T_) (counts) used to discriminate between NP and the background according to Equation (1) [15,18].

(1) SDL= (6 × 106 × ITπ × ρ × S × X)13

where *ρ* is the particle density (kg dm^−3^), *S* is the sensitivity (counts fg^−1^) and *X* is the fraction of measured element in the NP. Similarly, the size quantification limit (SQL) can be defined as the smallest diameters that can be detected with confidence. Thus, SDL is a mean value that is calculated from the sum of the threshold intensity (*I*_T_) and 3 times its standard deviation (SD), whereas the SQL was determined from *I*_T_ + 10SD.

A modified version of NuQuant (Nu Instruments, UK) was used for the treatment of the TOF-ICP-MS data. In that case, the algorithm searched for a target isotope, i.e., ^140^Ce, using similar smoothing and peak detection parameters as with SF-ICP-MS. Following detection of the Ce-containing peaks, each particle event was assigned start and end timestamps, which were used to integrate peaks for all other isotopes [37]. As above, the criteria to report peak events as NP were based on the FWHM values as well as the standard deviation of the background (for each isotope), which were used to estimate threshold values. Artifacts were flagged based upon abnormally large or small FWHM values. For the TOF-ICP-MS, thresholds were typically based upon 5–7 multiples of the standard deviation of the background, which were selected to remove most background artifacts, while optimizing the detection of the real NP peaks [37].

## 4. Conclusions

In conclusion, the use of a high sensitivity sector field ICP-MS with very short dwell times (50 µs) improved sensitivity (200× more) and decreased SDL (4× less) for Ce, when compared to the use of a Q-ICP-MS. The sensitivity and SDL were further improved when the SF-ICP-MS was coupled to a desolvator; however, this setup was shown to be much more sensitive to the presence of the dissolved analyte. SF-ICP-MS was shown to be useful to detect and characterize Ce-containing NPs in natural samples such as rain or river water samples. The use of a TOF-SP-ICP-MS allowed us to show that around a third of the nanoparticles in the Montreal rainwater were engineered CeO_2_ NPs.

## Figures and Tables

**Table 1:** Sensitivity for Ag, Au and Ce and the resulting size detection limits for Ag, Au and CeO_2_ NPs in Milli-Q water obtained using a quadrupole (Q-) or sector field (SF-) ICP-MS running with (dry) or without a desolvator (wet). A 50 µs dwell time was used. Means and standard deviations are obtained from analysis on three different dates.

Analyte	Sensitivity (Counts fg^−1^)			Nanoparticle	Size Detection Limit (nm)		
	Q-ICP-MS	SF-ICP-MS			Q-ICP-MS	SF-ICP-MS	
		Wet	Dry			Wet	Dry
**Ag**	50 ± 19	4750 ± 1200	22,300 ± 2400	**Ag**	16.3 ± 2.1	4.7 ± 0.2	3.1 ± 0.3
**Au**	15 ± 3	2900 ± 800	13,700 ± 1200	**Au**	19.0 ± 1.0	4.4 ± 0.1	2.8 ± 0.1
**Ce**	72 ± 15	14,800 ± 700	47,900 ± 8600	**CeO_2_**	16.8 ± 1.1	4.0 ± 0.2	2.3 ± 0.2

**Table 2:** Average particle sizes, number concentrations and SDL in 10 ng L^−1^ suspensions of Ag, Au and CeO_2_ NPs. Means and standard deviations are obtained from the analysis of triplicate samples.

NP Suspension	Average Particle Size (nm)		NP Number Concentration (×10^7^ L^−1^)		SDL (nm)	
	Wet	Dry	Wet	Dry	Wet	Dry
**Ag**	9.3 ± 0.4	8.5 ± 0.1	5.5 ± 0.6	7.7 ± 0.2	4.6 ± 0.1	3.1 ± 0.1
**Au**	11.8 ± 0.1	9.7 ± 0.1	6.6 ± 0.8	8.9 ± 0.1	4.2 ± 0.1	3.1 ± 0.1
**CeO_2_**	8.0 ± 0.1	6.3 ± 0.0	20.9 ± 0.4	26.3 ± 1.5	4.1 ± 0.1	2.8 ± 0.1

**Table 3:** Concentration of dissolved Ce, number and mass concentrations of CeO_2_ NPs and SDL in the rainwater and in the St. Lawrence River. Means and standard deviations are obtained from the analysis of triplicate samples.

Water Natural Sample	Mass Concentration of Dissolved Ce (ng L^−1^)	Number Concentration (×10^9^ CeO_2_ NPs L^−1^)	Mass Concentration of CeO_2_ NPs (ng L^−1^)	SDL (nm)
**Montreal rainwater**	4.0 ± 0.1	0.22 ± 0.01	1.9 ±0.1	4.6 ± 0.1
**St. Lawrence River**	53 ± 17	1.6 ± 0.3	107 ± 26	6.2 ± 0.6

**Table 4:** Ratios of Ce to La determined in the Montreal rainwater, obtained using the SF-ICP-MS run in single particle mode with a dwell time of 50 µs. Means and standard deviations were obtained from triplicate samples.

Water Natural Sample	Fraction of Ce NPs (%)	Fraction of La NPs (%)	Ce NPs/La NPs	Dissolved Ce/Dissolved La	Total Ce/Total La
**Montreal rainwater**	46.3 ± 8.8	50.6 ± 3.9	2.2 ± 0.6	2.6 ± 1.8	2.4 ± 2.2

## Supplementary Materials

The following are available online, **Figure S1**: Time resolved signal of ^140^Ce in a suspension of 10 ng L^−1^ CeO_2_ NPs spiked with 10 ng L^−1^ of ionic Ce measured using different dwell times. **Figure S2.** Particle size distributions of a 10 ng L^−1^ suspension of CeO_2_ NPs with a nominal size range 1–10 nm spiked with ionic Ce. **Figure S3.** Time-resolved signal for ^140^Ce in a 10 ng L^−1^ suspension of CeO_2_ NPs with a nominal size range 1–10 nm spiked with ionic Ce. **Figure S4.** Size detection limit as a function of concentration of dissolved Ce, as measured by wet-SF-ICP-MS and dry-SF-ICP-MS. **Figure S5.** Time-resolved signal for ^140^Ce in **(A)** unfiltered rainwater and **(B)** unfiltered St. Lawrence River water. **Figure S6.** Particle size distributions of Ce containing NPs in St. Lawrence River water with or without filtration. **Table S1.** Mean particle sizes, NPs number concentrations, mass concentration of dissolved Ce and SDL for Ce containing NPs in St. Lawrence River water, with or without filtration. **Figure S7.** Sampling locations on the St. Lawrence River. **Figure S8.** Time-resolved signal for **(A)** ^140^Ce and **(B)** ^139^La in Montreal rainwater. **Table S2.** Mean particle sizes, NPs number concentrations and mass concentration of dissolved and nanoparticulate Ce and La in Montreal rainwater. **Figure S9.** Time-resolved signal for ^139^La (red) and ^140^Ce (blue) in Montreal rainwater analyzed by TOF-SP-ICPMS.
