# Supplementary material for: Measurement of CeO2 Nanoparticles in Natural Waters Using a High Sensitivity, Single Particle ICP-MS
Source: Molecules. 2020 Nov 25;25(23):5516. doi: 10.3390/molecules25235516 (PMC7734582; doi:10.3390/molecules25235516)
Supplement: Supplementary file 1 [file molecules-25-05516-s001.pdf]

Supplementary Materials

Measuring CeO_2 nanoparticles in natural samples using a high sensitivity sector field single particle ICP-MS.

Submitted to:

Trends in ICP–MS Analysis - From New Methods to Recent Applications

IBRAHIM JREIJE[1], AGIL AZIMZADA[1,2], MADJID HADIOUI[1], KEVIN J. WILKINSON[1].

[1]Biophysical Environmental Chemistry Group, University of Montreal, P.O. Box 6128, Succ. Centre-Ville, Montreal, QC, Canada.

[2]Department of Chemical Engineering, McGill University, Montreal, QC, Canada.

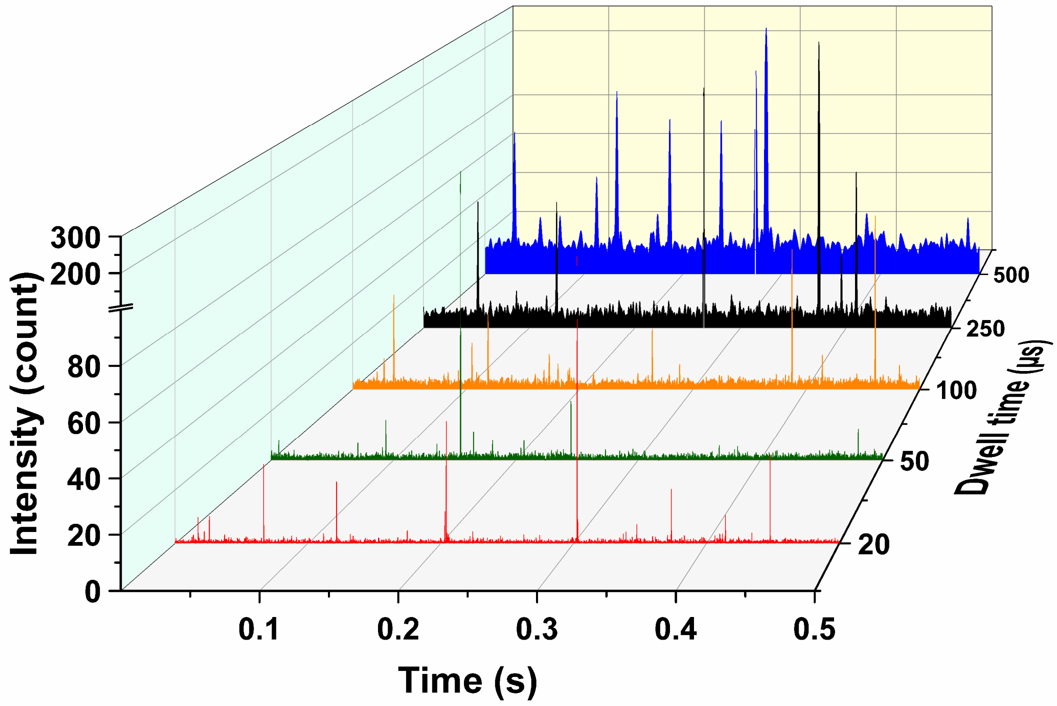

Figure S1. Time resolved signal of ^{140}Ce in a suspension of 10 ng L^{-1} CeO_2 NPs spiked with 10 ng L^{-1} of ionic Ce measured using different dwell times. Measurements were obtained using dry-SF-ICP-MS.

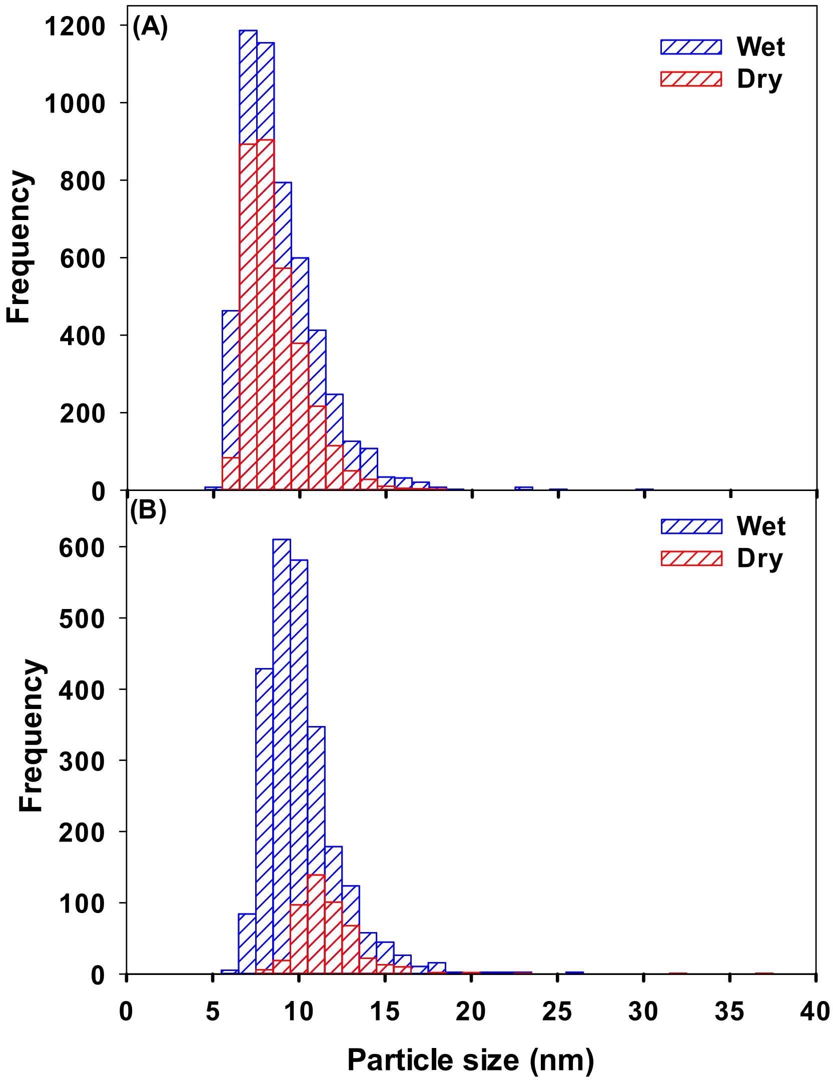

Figure S2. Particle size distributions of a 10 ng L^{-1} suspension of CeO_2 NPs with a nominal size range 1-10 nm spiked with ionic Ce at a mass concentration of **(A)** 10 ng L^{-1}; **(B)** 50 ng L^{-1} as measured by Wet-SF-ICPMS (blue) and Dry-SF-ICPMS (red). The mean particle size ± the standard deviation on the distribution in the suspension in **(A)** is 8.3 ± 2.3 with dry introduction and 8.1 ± 1.8 with wet introduction; **(B)** is 11.2 ± 2.3 with dry introduction and 9.7 ± 3.2 with wet introduction.

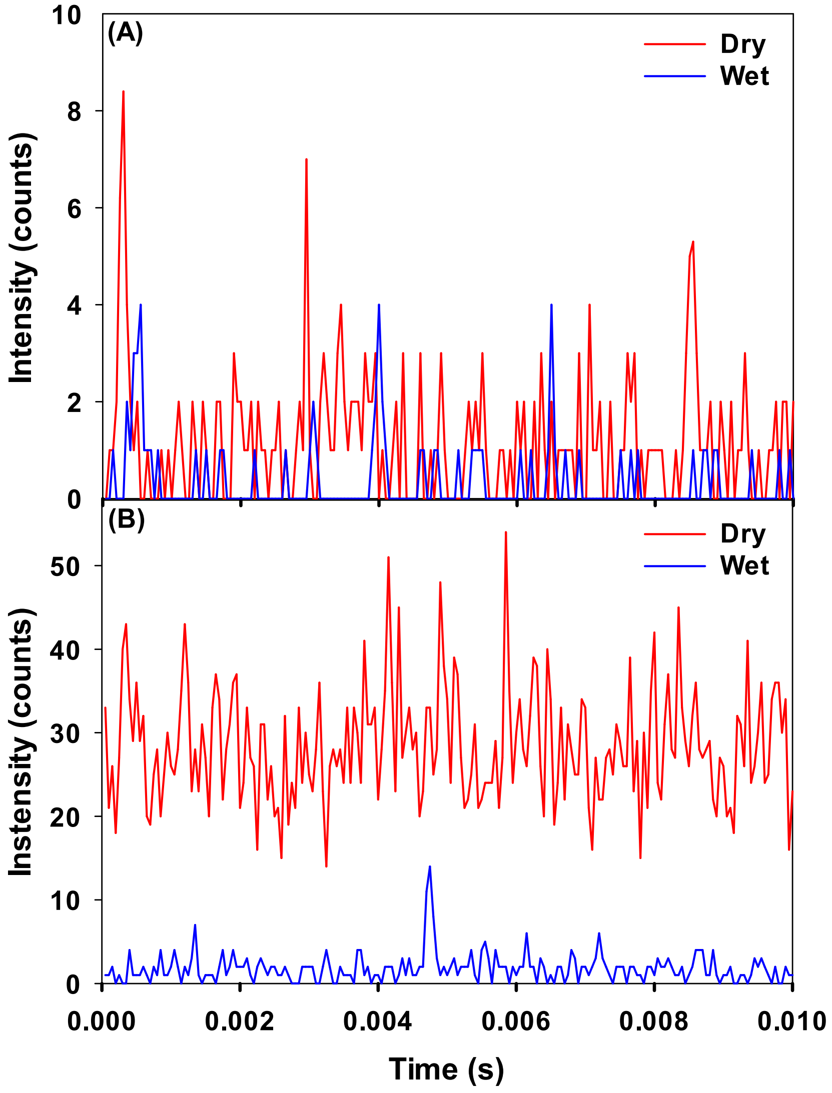

Figure S3. Time-resolved signal for ^{140}Ce in a 10 ng L^{-1} suspension of CeO_2 NPs with a nominal size range 1-10 nm spiked with ionic Ce at a mass concentration of **(A)** 10 ng L^{-1}; **(B)** 50 ng L^{-1} as measured by Wet-SF-ICPMS (blue) and Dry-SF-ICPMS (red). For better readability, only 0.01 s of data collection were shown, the total acquisition time was 50 s. A dwell time of 50 µs was used with both introduction systems.

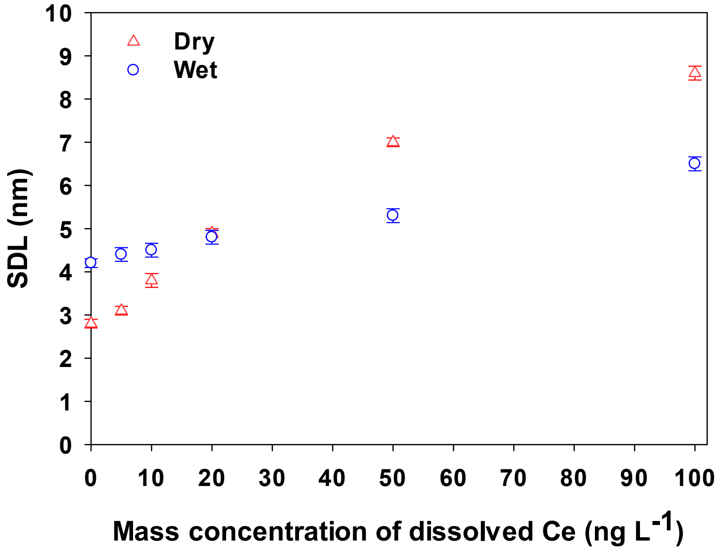

Figure S4. Size detection limit as a function of concentration of dissolved Ce, as measured by wet-SF-ICP-MS (blue) and dry-SF-ICP-MS (red). A dwell time of 50 μs was used with the two introduction systems.

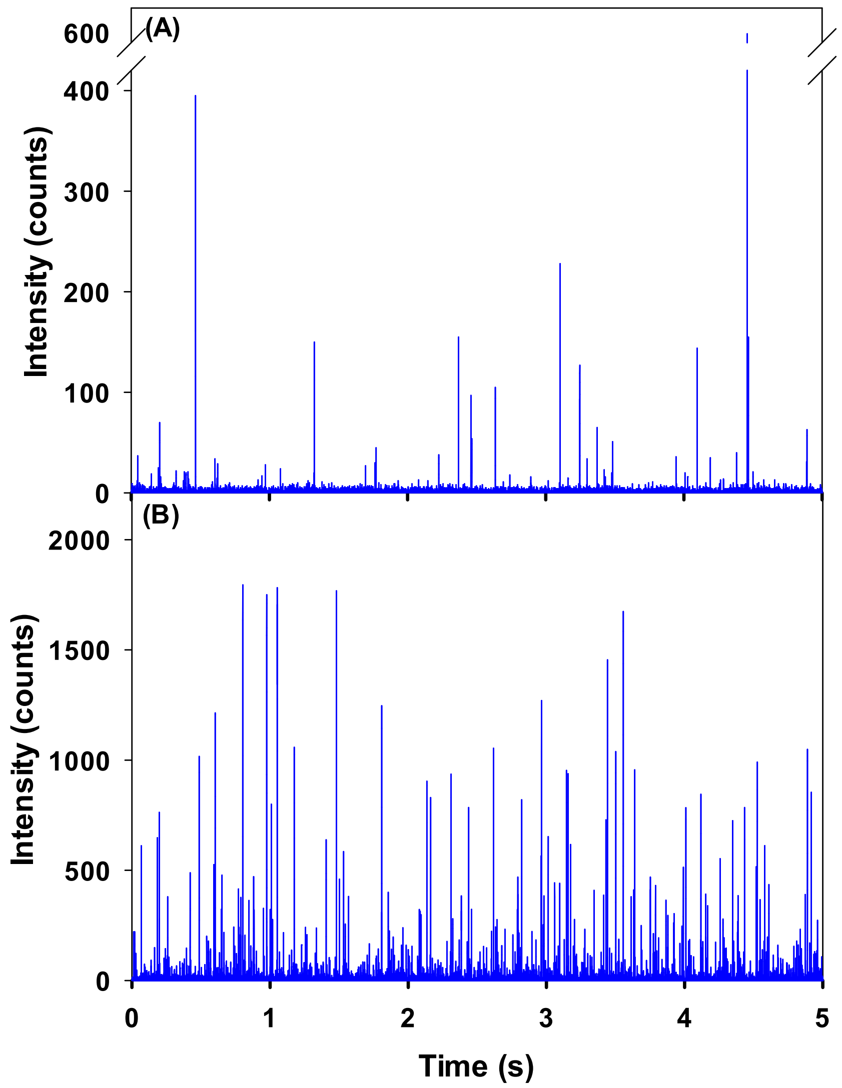

Figure S5. Time-resolved signal for ^{140}Ce in **(A)** unfiltered rainwater and **(B)** unfiltered St. Lawrence River water. For better readability, only 5 s of data collection were shown, the total acquisition time was 50 s. Measurements were obtained using the wet-SF-ICP-MS with a 50 µs dwell time.

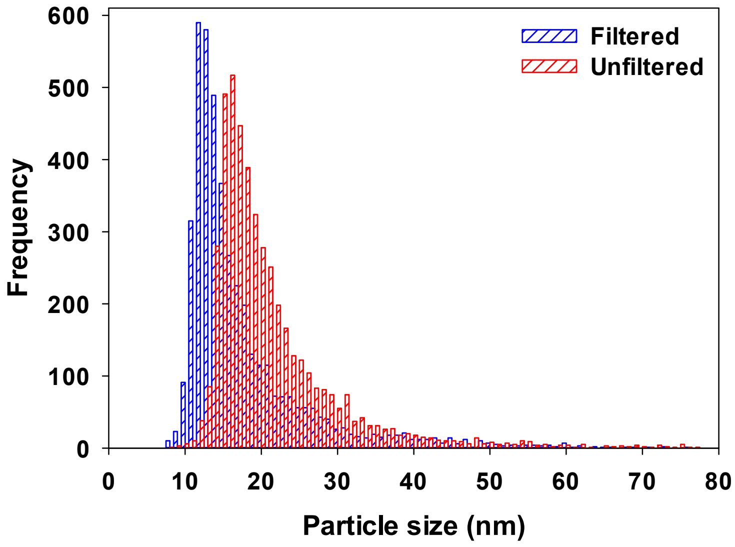

Figure S6. Particle size distributions of Ce containing NPs in St. Lawrence River water with (blue) or without (red) filtration. Samples were filtered using syringe filters with a PVDF membrane with a 0.45 µm poresize. NP sizes were calculated by assuming that the particles were spherical CeO_2 particles with a density of 7.13 kg dm^{-3}. Measurements were obtained using the wet-SF-ICP-MS with a 50 µs dwell time.

Table S1. Mean particle sizes, NPs number concentrations, mass concentration of dissolved Ce and SDL for Ce containing NPs in St. Lawrence River water, with or without filtration. Measurements were obtained using the wet-SF-ICP-MS with a 50 µs dwell time. Means and standard deviations are obtained from triplicate samples.

St. Lawrence River	Mean particle size (nm)	Number concentration (x 10^9 CeO_2 NPs L^{-1})	Mass concentration of dissolved Ce (ng L^{-1})	SDL (nm)
**Filtered**	17.2 ± 0.0	1.13 ± 0.02	36.0 ± 0.6	5.2 ± 0.1
**Unfiltered**	21.9 ± 0.9	1.60 ± 0.32	52.7 ± 17.0	6.2 ± 0.6

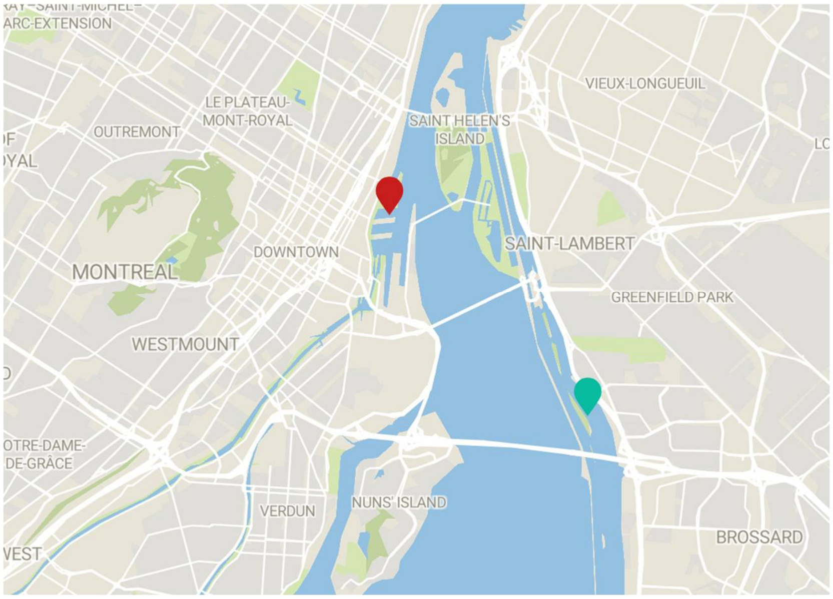

Figure S7. Sampling locations on the St. Lawrence River. The green mark corresponds to the location (coordinates: 45.477473, -73.504144) where the results of the sample were presented in **Figure 4**, **S4** & **S5**; the red mark corresponds to the location (coordinates: 45.5090203, -73.5485275) of the samples used for the temporal variability experiments (**Figure 5**).

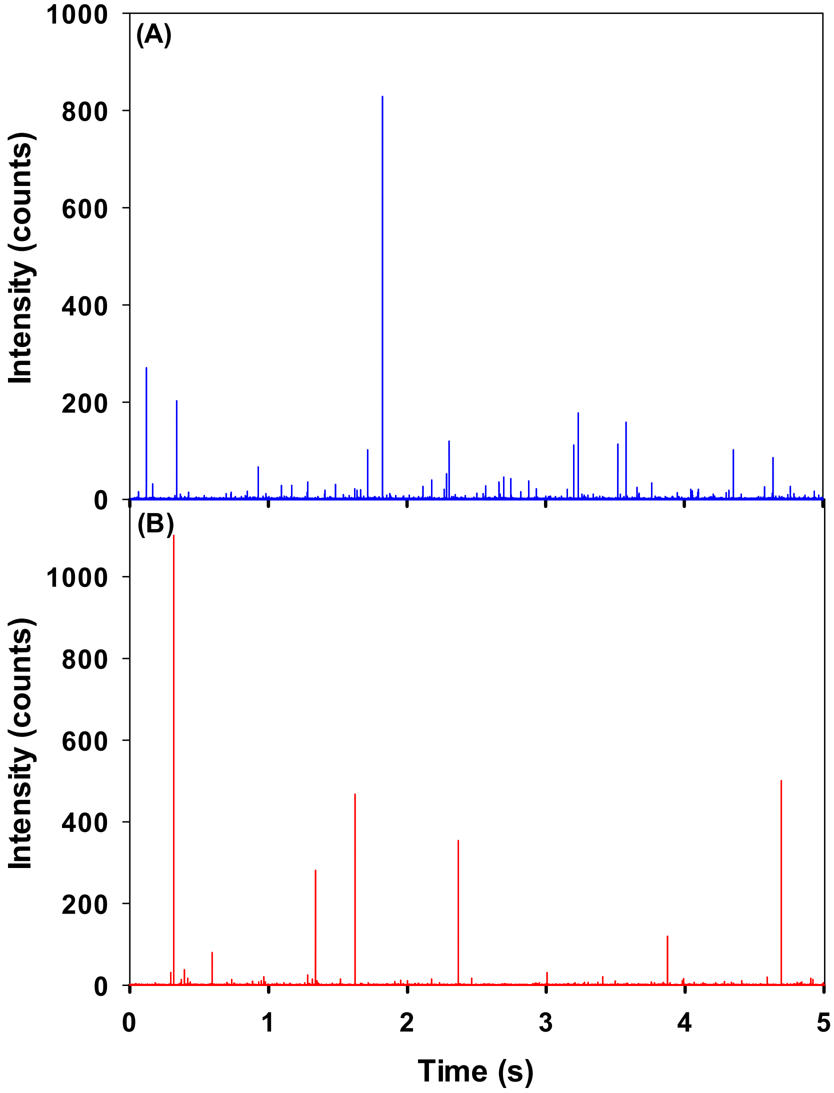

Figure S8. Time-resolved signal for **(A)** ^{140}Ce and **(B)** ^{139}La in Montreal rainwater. For better readability, only 5 s of data collection were shown, the total acquisition time was 50 s. Measurements were obtained using the wet-SF-ICP-MS with a 50 µs dwell time.

Table S2. Mean particle sizes, NPs number concentrations and mass concentration of dissolved and nanoparticulate Ce and La in Montreal rainwater. Measurements were obtained using the wet-SF-ICP-MS with a 50 µs dwell time. Means and standard deviations are obtained from triplicate samples.

Water natural sample	Mean particle size (nm)		Number concentration (x 10^8 L^{-1})		Mass concentration of dissolved (ng L^{-1})		NPs mass concentration (ng L^{-1})	
	CeO_2	La_2O_3	Ce	La	Ce	La	Ce	La
**Montreal rainwater**	10.0 ± 1.1	9.9 ± 0.4	3.9 ± 0.5	1.6 ± 0.6	2.4 ± 0.8	1.0 ± 0.4	2.0 ± 0.1	1.0 ± 0.2

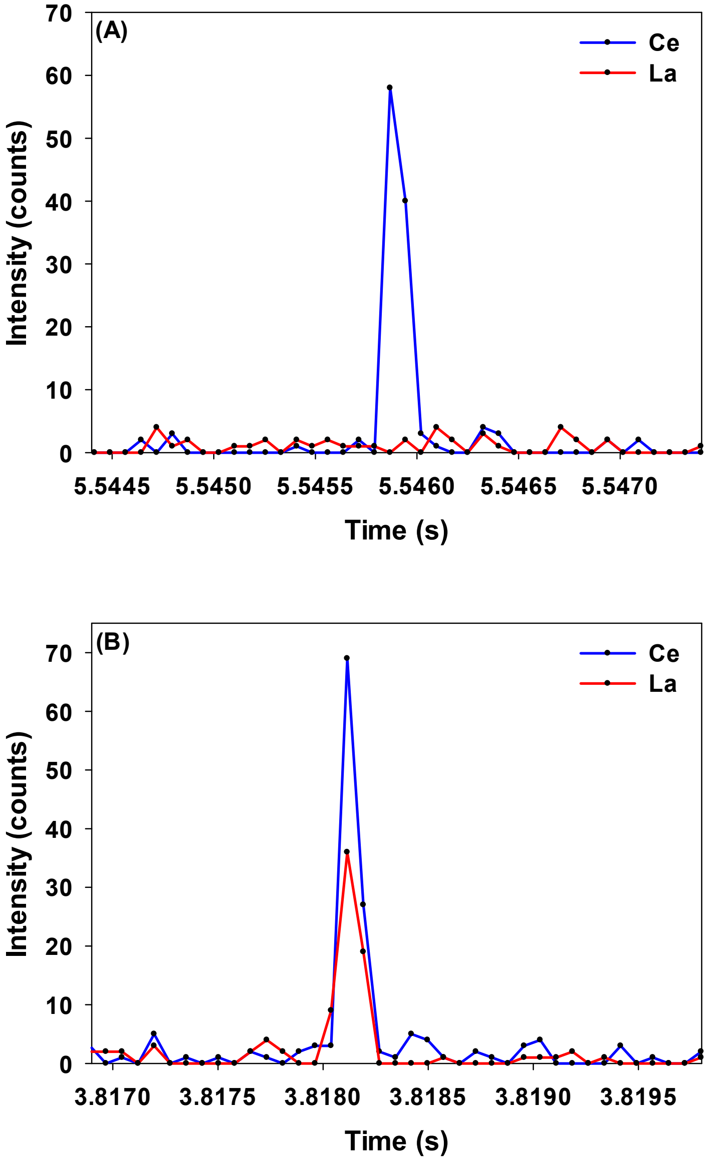

Figure S9. Time-resolved signal for ^{139}La (red) and ^{140}Ce (blue) in Montreal rainwater analyzed by TOF-SP-ICPMS showing **(A)** a pure Ce NP and **(B)** a NP that contains Ce and La.
